# Neurocognitive Profile and Associated Factors Among Children Affected by Sickle Cell Disease in Kinshasa, Democratic Republic of Congo: A Cross-Sectional Study

**DOI:** 10.3390/children11121521

**Published:** 2024-12-14

**Authors:** Patricia V. M. Lelo, Faustin Nd Kitetele, Marcel Kunyu, Cathy E. Akele, Daniel L. Okitundu, David Lackland Sam, Michael J. Boivin, Espérance Kashala-Abotnes

**Affiliations:** 1 Department of Infectious Diseases, Kalembelembe Pediatric Hospital, Kinshasa 012, Democratic Republic of the Congo; fnd023@uib.no (F.N.K.); akelekat@yahoo.fr (C.E.A.); 2 Centre for International Health (CIH), Faculty of Medicine, University of Bergen, 5020 Bergen, Norway; david.sam@uib.no (D.L.S.); ekashala@uottawa.ca (E.K.-A.); 3 Department of Neurology, University of Kinshasa, Kinshasa 012, Democratic Republic of the Congo; klmarcel@yahoo.fr (M.K.); daniel.okitundu@unikin.ac.cd (D.L.O.); 4 Department of Psychiatry and Department of Neurology & Ophthalmology, Michigan State University, East Lansing, MI 48824, USA; boivin@msu.edu; 5 Department of Psychiatry, The University of Michigan, Ann Arbor, MI 48109, USA; 6 Department of Psychiatry, Faculty of Medicine, University of Ottawa, Children Hospital of Eastern Ontario, Ottawa, ON K1J 9B7, Canada

**Keywords:** sickle cell disease, Mullen Scales of Early Learning, early child neurodevelopment and cognition, Gensini Gavito Scale, Democratic Republic of Congo

## Abstract

Background/Objectives: Understanding the neurocognitive profile of children with sickle cell disease in the Democratic Republic of Congo is essential, as this condition can significantly affect their development. Our study aims to assess these children’s neurocognitive and developmental profiles and identify related factors. Methods: We conducted a descriptive cross-sectional study involving 287 children, aged 0 to 68 months, using the Mullen Scales of Early Learning and the Gensini Gavito Scale. We also screened for maternal depression using the Hopkins Symptoms Checklist-10. Results: More than half of the participants were boys, with an average age of 4 years. Remarkably, 95.8% (score T < $\bar{x}$ +2 SD) of children scored below average on the Mullen Scales. Significant associations were found between early neurocognitive development and factors like maternal depression, socioeconomic status, maternal education, age of weaning, and responses to the Ten-Questions Questionnaire (*p* < 0.005). Conclusion, children with sickle cell disease show below-average cognitive development, with maternal depression being a critical factor. Longitudinal studies are vital to understanding the long-term cognitive effects of sickle cell disease, particularly in the Democratic Republic of Congo, where targeted support is urgently needed.

## 1. Introduction

Sickle cell disease (SCD) is one of the world’s most common haemoglobinopathies [1], and it is a disease that presents very severe symptoms in affected children [2]. Around the world, SCD is estimated to affect more than 400,000 newborns annually, with 80% from low- and middle-income countries [1]. The disease is caused by a single-point mutation in the beta-globin gene, which results in the formation of sickle haemoglobin (HbS) [3]. Under certain conditions, including hypoxia, HbS polymerises and creates distorted (i.e., ‘sickle’-shaped), adherent, and less deformable red blood cells (RBCs) [4]. The result is easily haemolyzed RBCs with a shortened lifespan, endothelial damage, vessel obstruction, and other pathophysiological effects that collectively contribute to the development of a vast constellation of acute and chronic clinical manifestations and, often, premature mortality [1]. Fetal haemoglobin (HbF), the predominant haemoglobin during gestation and in neonates, is the most potent known inhibitor of HbS polymerization. As such, infants with SCD are asymptomatic until HbF levels decline, typically within the first 6–24 months of life. Early diagnosis prior to the predominance of HbS is critical to allow for the provision of early lifesaving interventions [1].

In Africa, it is estimated that 200,000 children are born with SCD every year [2]. The prevalence of SCD is notably high across the continent, where in some regions more than 2% of births are reported to be affected, contributing silently but significantly (8–16%) to the mortality of those under 5 years of age in high-burden countries [1]. Because of SCD, the mortality of children remains very high. Only one in five children will reach the age of five, while in Europe and America, more than 90% of children will reach adulthood. This difference depends on the climate, environment, socioeconomic conditions, and, mainly, on the national health policies adopted in these countries [4,5]. Unfortunately, SCD is growing; it is estimated that 14 million children will be born with the homozygous form of SCD, with over 70% of these births occurring by 2050 [4,6].

The Democratic Republic of Congo is the third country most affected by SCD [3,7]. It is the second largest country in Africa, with a high prevalence of SCD. The haemoglobin subunit beta gene (HBB) allele frequency in neonates varies across the country, from 0.96% to 1.4% [8,9,10]. According to the National Program for SCD Control (PNLCD), SCD is estimated to affect about 3% of the Congolese population in general, and 12% of children hospitalized in paediatric wards have SCD [11].

Patients affected by SCD and their families are constantly fighting against the disease and lobbying the health authorities to improve the quality of life and life expectancy of people with SCD. The country has implemented national policies and strategies to improve care and combat SCD. Currently, newborn screening and oxycarbamide treatment (hydroxyurea) are available for children with SCD. Other emerging and promising therapies are on the horizon, like bone marrow transplantation [4].

SCD is a haemolytic disease that causes chronic anemia, leading to hypoxia, which affects the whole body, including organs. One consequence for the nervous system is noticeable cognitive deficits and psychological disturbances due to chronic anemia and hypoxia, which can lead to brain damage and recurrent microinfarctions [2,12,13].

The cumulative effect of brain damage is likely to result in compromised early childhood neurodevelopment and cognitive functioning [14]. The brain’s most affected functions resulting from chronic hypoxia are the attention and executive functions [15]. These neurocognitive impairments are already present early in life [4,16].

In the Democratic Republic of Congo, all children are treated with the standard treatment comprising folic acid, penicillin V, and hydroxyurea. Hydroxyurea is one of the main treatments that can slow down sickness progression in patients with SCD. However, its high-cost limits access for many patients. [17,18]. This standard treatment in the DRC recommends anti-bacterial prophylaxis against pneumococcal infections. We also recommend the following vaccines: Pneumococcal, haemophilus influenza, Meningococcal, Hepatitis A, Hepatitis B, and typhoid, according to the national schedule [18]. Concerning medical care, studies have shown that success in SCD management follows a comprehensive approach that is focused on patients and their families [19].

The Mullen Scales of Early Learning (MSEL) tool has been successfully applied in various African nations to assess early childhood neurodevelopment and cognitive skills. Positive outcomes have been seen in countries like Benin [20], West Africa [21], Cameroon [22], Tanzania [12], and Uganda [23], as well as in children affected by Konzo disease in the DRC [24].

To the best of our knowledge, there are few available data on cognition in children affected by SCD. The studies that are available looked at children aged 6–18 years old [25]; there are no available data on very young children in sub-Saharan Africa in general, and in the DRC in particular. There is a definite need to better understand the neurocognitive profile of younger children affected by SCD to ensure and promote healthy lives and well-being for all children as recommended by Sustainable Development Goal 3 (SDG Health) [26]. We therefore aimed to describe the neurocognitive profile and early childhood development of children with SCD and identify the associated risk factors among children with SCD in Kinshasa, Democratic Republic of Congo.

## 2. Methods

### 2.1. Study Site

The present study was conducted across three strategically selected hospitals in Kinshasa, the capital of the DRC, which provide specialized services for managing sickle cell disease (SCD).

The first institution, Kalembelembe Pediatric Hospital (KLPH), is notable for being the only paediatric hospital in the country. It was established in 1948 through a collaborative effort between the Belgian Red Cross and the DRC Ministry of Health. KLPH focuses on the healthcare needs of children aged 0 to 15. In addition to its expertise in SCD management, KLPH offers children a range of paediatric services [27,28].

The second facility, Mabanga (Centre for Mixed Medicine and Anemia SS or CMMASS), occupies a prominent position as the official institution in the DRC specializing in the management of SCD. Historically, it was the first hospital established in Central Africa specifically for treating patients with SCD, underscoring its longstanding commitment and significant role in advancing care within this specialty [28].

The third, Monkole Hospital, is a private referral facility established in 1991. The hospital collaborates with the Ministry of Health in the Democratic Republic of Congo. It has developed a specialized unit focused on the comprehensive management of patients with sickle cell disease (SCD). This initiative enhances the capacity for providing high-quality care within the healthcare system. It also has representation in some rural areas [28].

These hospitals were selected for their expertise in managing SCD. They form a robust network of care that effectively addresses the needs of individuals with SCD in Kinshasa, its surrounding provinces, and neighboring countries. This selection underscores their importance as vital healthcare resources, significantly enhancing the overall health landscape in the DRC. By focusing on their commitment and capacity to treat those patients, these hospitals play a crucial role in expanding the study’s reach and impact, ultimately benefiting a larger community.

### 2.2. Study Population

Our study included all children aged between 0 and 68 months who were being followed up at these three selected hospitals.

### 2.3. Study Design

We conducted a cross-sectional descriptive study to describe the neurocognitive profile and early child neurodevelopment of children aged 0 to 68 months with SCD in three selected paediatric hospitals in Kinshasa/DR Congo. The study took place between May 2018 and February 2020. The Mullen Scales of Early Learning (MSEL) and the Gensini Gavito Scale assessed the children’s early neurodevelopment and cognitive skills. These assessment tools have shown strong efficacy in previous research conducted in the DRC [24]. Each child in our study underwent comprehensive clinical and neurological evaluations, complemented by a specially designed questionnaire that gathered essential information about their socioeconomic status. In our commitment to ethical research practices and to ensure the well-being of our participants, children displaying clinical symptoms, such as fever, abdominal pain, bone pain, or dyspnea, were identified and not assessed on the evaluation day. They received appropriate medical attention and were rescheduled for a new assessment appointment. This approach reflects our commitment to the health of the children involved in our study.

### 2.4. Inclusion Criteria

All children who met the following criteria were included in the study:

(i) Confirmed to have SCD, either by the Hb electrophoresis technique, Sickle Rapid Scan test, or by both methods, as documented in the special SCD clinic card held by each patient;
(ii) Aged between 0 and 68 months old who were regularly followed up at either of the three selected hospitals;
(iii) Clinically stable during the assessment;
(iv) Whose parents or caregivers provided informed consent to participate in the study.

### 2.5. Exclusion Criteria

All children presenting with any acute condition that would limit their ability to participate in the study were excluded.

### 2.6. Study Procedure

#### 2.6.1. Sociodemographic, Economic, and Home Environment Covariates

Nurses conducted structured interviews with the parents/caregivers on the same day in the hospital, gathering information on the sociodemographic, socioeconomic, and home environment before the children’s medical visit. The parental level of education was scaled from 0 (no education) to 4 (university level). To assess socioeconomic status, we ranked the status of individuals using a generated wealth index based on the assets and housing quality (type of floor, roofing, toilet facilities, water source, electricity, etc.). This method has been used previously in the DRC [24]. The index is constructed as follows: (1) Each good or characteristic is assigned a point (score or coefficient) generated from a principal component analysis. (2) The resulting good scores are standardized according to a standard normal distribution with a mean of 0 and standard deviation of 1. (3) Each household is assigned a score for each good, and all household scores are summed. (4) Households are ranked in ascending order according to the total score and divided into three categories of equal size called tertiles (i.e., lowest tertile, middle tertile, and highest tertile). (5) Households are, thus, allocated to the different categories [24,29]. We used the short version of the Caldwell Home Observation for Measurement of the Environment (Home), adapted to the African context [20,24], to assess the children’s stimulation level and the learning opportunities the family environment offers.

We interviewed parents/caregivers using the 18 items related to the level of stimulation and learning opportunities provided to the child.

#### 2.6.2. Child and Mother Health Covariates

All children were clinically examined by a physician, including a neurological examination and anthropometric measurements taken according to standard procedures. As per the WHO’s recommendations, mid-upper arm circumference (MUAC) was used as a standard measurement of nutritional status [30]. Maternal depression and anxiety symptoms were assessed through a structured interview using the Hopkins Symptoms Checklist-10 (HSCL-10).

HSCL-25 is a well-known and widely used screening instrument for anxiety and depression [24,31,32]. We used the short version of the HSCL-10, considered a good screening instrument in primary healthcare settings and research [24,32]. The HSCL-10 is an easy-to-administer scale that has been validated in the DRC [24]. All derived scores from the HSCL-10 were analyzed as continuous variables.

#### 2.6.3. Measure of Early Child Neurodevelopment and Neurocognitive Outcomes

Early child development and cognition were measured through clinical observation and interviews with parents/caregivers. The tools selected to measure early child development and cognition in the present study are presented below.

The Mullen Scales of Early Learning (MSEL) is easy to administer and useful for assessing early child neurodevelopment and cognition in low-resource settings. It is a quick and reliable assessment tool for childhood development, measuring cognitive ability and motor development [24,33]. We used a French version of the MSEL, and the instructions were explained in French and the local language. The study investigator (P. L.) was trained by a psychologist (M. J. B.), and the rest of the study team was trained and supervised by a neuropsychiatrist. The MSEL has already been used in the DRC among children affected by Konzo, a neurological disease [24], and has also been validated and used in several African studies [19,24].

The Gensini Gavito Scale (GGS) is a neurodevelopmental scale to assess children’s psychomotor development and growth in low-income settings. The scale is widely used, adapted locally, and validated for the DRC [24,34]. It was selected to measure early child neurodevelopment, cognition, and psychomotor development. The scale generates a psychomotor developmental quotient (PDQ) expressed in percentage. The PDQ is the ratio of the child’s actual age (RA) to their acquisitions in the following three domains: motricity, perception and communication, and adaptation. It is calculated as the child’s chronological age (CA) at the time of the examination times 100, as follows: PDQ = (RA/CA × 100). The scale is widely used in paediatric clinics in Kinshasa among children affected by malaria [35]. The PDQ reported was used as a continuous variable in the analysis.

The Ten-Question Questionnaire (TQQ) [36] is a screening tool for child disability developed for use in resource-limited settings. It gathers information on child development and disabilities as perceived by the mother. It is the most widely used tool for child disability assessment in (LMICs) [37,38] and has already been used in the DRC [24]

Physicians (K. L., M. K., and P. L.) supervised all clinical evaluations and gathered medical information on pregnancy, milestones, growth, and breastfeeding practices. Each child was assessed using a standardized format in the same context and the presence of the primary caregiver.

### 2.7. Data Collection

#### Recruitment

A clinical registry was used to identify cases. One month before the study began, health workers visited parents/guardians to inform them about the study and obtain consent. To be reassured of their attendance, the nurse reached out by phone one day before their scheduled appointment to those who had given verbal consent, serving as a reminder. Each parent’s/guardian’s telephone number was recorded in the patient’s file. Each parent/guardian signed the informed consent forms on the evaluation day. During the assessment of the child, parents or caregivers were seated in a chair positioned behind the child. The child and the examiner were seated at the test table appropriately adjusted to the child’s height. If a child left their seat during the assessment, they were gently encouraged to return. If the child faced challenges in doing so, the examiner patiently waited for them to come back to the table. Part of the assessment was also conducted on the ground until the child finally returned to the test table. Some children occasionally needed to take a long break of up to 30 min during the assessment. This approach ensured the environment was supportive and accommodating, ultimately leading to a more comfortable experience for the children involved.

### 2.8. Statistical Analyses

Early child neurodevelopment and cognitive ability were the main outcomes of our study. The data for the study, collected on a paper-based questionnaire, were encoded in Excel 2016. The questionnaire was written in French, and after quality control and consistency checking of the database, the data were exported to SPSS 23.0 and Stata 14 for analysis. The means and their standard deviations were calculated for normally distributed continuous variables, while the proportions with their 95% confidence intervals were calculated for the categorical variables. The median was calculated for continuous variables with an asymmetric distribution. We calculated the mean T-scores of the sample for all scales of the MSEL. All children with a T-score below the mean plus 2 SD were considered below average. We identified the proportion of children with a composite score < $\bar{x}+2\;SD$.

For the composite variables (principal component analysis), we used the Cronbach’s alpha coefficient, an index between 0 and 1, to assess the homogeneity of a set of items: the socioeconomic level. We considered the instrument’s homogeneity satisfactory when the coefficient’s value was at least equal to 0.70.

Multiple linear regression was used to identify significant associated factors. Two models were estimated with the following two dependent variables: the Mullen score for early learning and the Gensini Gavito score. The variables introduced in this model were the household socioeconomic level, nutrition, food security, and maternal mental health, as measured by the Hopkins Symptoms Checklist (HSCL-10). It also considers additional elements such as the TTQ, education, educational style, environment, and the impact of the QPFPF. A significance threshold of α = 0.05 was established for all tests to ensure an accurate analysis. Analyses of the nutritional status of children with SCD were carried out using the WHO’s WhoAnthro application (https://www.who.int/tools/child-growth-standards/software, accessed on 9 December 2024).

### 2.9. Ethical Approval

Ethical approval was obtained from the DRC National Health Ethics Committee at the Ministry of Health (code: 61/CNES/BN/PMMF/2018) and the Norwegian Regional Ethics Committee for Medical and Health Research (code: 2018/581-1). The purpose and nature of the study were explained to all parents. Written informed consent was obtained from all parents (caregivers/guardians) of children with SCD involved in the study, and parental permission was also obtained for each child.

## 3. Results

### 3.1. Sample Characteristics

A total of 287 children were included in the study. The children’s mean age was 4 years old (min. = 0; max. = 68 months old). The were 154 (54%) boys and 133 (46.3) girls. The majority of the children, 271 (94%), were born in the region, and all children included in the study resided in Kinshasa and had been doing so for more than six months before the study. Almost 66% of the children were out of school, highlighting an important area for potential intervention. Most caregivers were mothers, 218 (75.9%), and most children, 220 (76.7%), lived with both parents. It was also reported that almost all children were exclusively breastfed until the age of 6 months and weaned before the age of 2 years. The mean birthweight was 3274.8 g (Table 1). All children (100%) in the study were on preventive folic acid and penicillin V treatment, which are critical for their health. Only eight children (2.8%) were on hydroxyurea treatment, indicating a focused approach to managing specific health needs.

### 3.2. Sociodemographic, Socioeconomic, and Home Environment Characteristics

These characteristics are presented in Table 1 and Table 2. The parents had varied educational backgrounds based on sex. Notably, 156 mothers (55.1%) had attained a secondary level of education, while 114 fathers (39.7%) possessed a university degree. Many of the parents were self-employed, with 52.3% of fathers and 48.8% of mothers engaged in entrepreneurial activities. Regarding health, it is important to recognize that 78 households (27.2%) had family members affected by sickle cell disease (SCD). Concerning nutrition, 22.6% of children were introduced to solid foods before 6 months. Importantly, the study indicated that alcohol consumption and smoking were uncommon among caregivers during pregnancy. More than half of caregivers, 167 (58.2%), reported using stimulation and learning opportunities such as storytelling for their kids. Very few parents, 9 (3.1%), reported leaving their children home alone more than 10 times/month. A good proportion of children played with bought toys, 274 (95.5%), and homemade toys, 229 (79.8%). Spanking was the common mode of punishment, 217 (75.6%), and a few parents, 11 (3.8%), used a stick to punish.

### 3.3. Child and Maternal Health

The child and maternal health characteristics are presented in Table 1. The findings indicate that most children in the study demonstrated healthy neurological profiles with no significant clinical signs of deficits. While parents reported that 13 children (4.5%) experienced neurodevelopmental delays in areas such as walking, language, and adaptation, these cases appear to be limited. Additionally, a small number of children exhibited neurological complications stemming from their sickle cell disease (SCD), with five children showing asymmetrical spastic gait. Two children required walking assistance, suggesting a targeted area for support and intervention. Importantly, most children underwent normal neurological examinations, with 98.3% exhibiting an appropriate gait, 97.6% showing normal muscle tone, 99% having proper reflexes, and 96.9% demonstrating adequate language development. Furthermore, no sensory issues were identified, underscoring the overall positive neurological health of the children. This information provides valuable insights for ongoing monitoring and potential early interventions to support the affected children.

**Figure 1:**
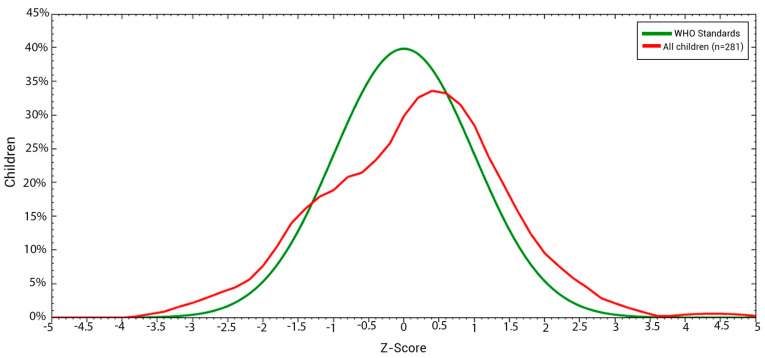
Nutritional status based on the weight-age index of the studied children aged ≤68 months in Kinshasa, DRC.

This graph illustrates the overall trend in children’s nutritional status based on the weight-for-age index, comparing it to the reference values set by the WHO standard. Our findings indicate that most children are classified as having a normal nutritional status according to their weight range for their age (Figure 1).

**Figure 2:**
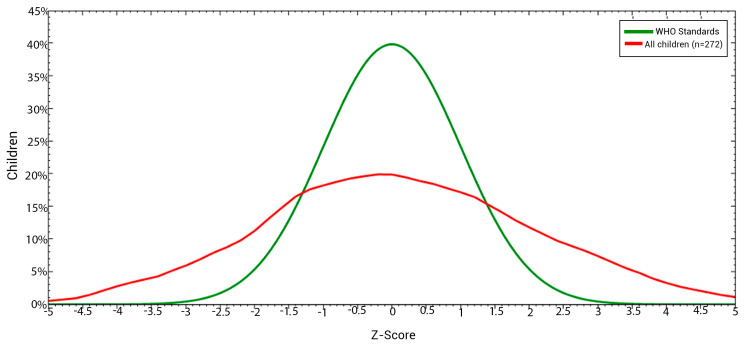
Nutritional status based on the height–age index of the studied children aged ≤68months in Kinshasa, DRC.

This graph highlights the range of growth patterns observed in children. It shows the proportion of normal children with good nutritional status based on the height-to-age index. Additionally, it emphasizes the following two extremes: at the one end, children facing severe growth retardation and malnutrition, and at the other, those experiencing gigantism with significantly elevated height-to-age ratios (Figure 2).

**Figure 3:**
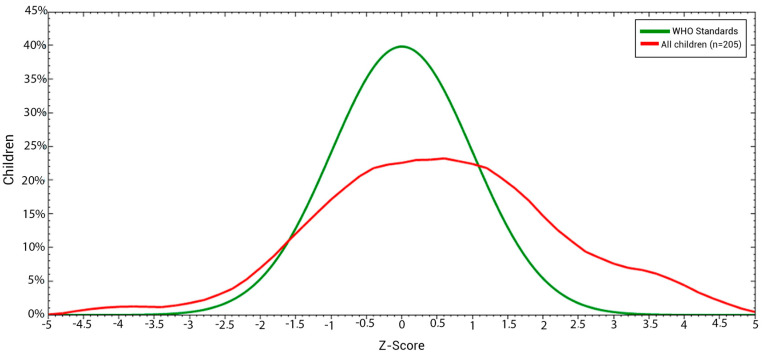
Nutritional status based on the weight–height index in z-scores, compared to the WHO’s standards, of the studied children aged ≤68 months in Kinshasa, DRC.

This graph illustrates that the distribution curve of the weight-to-height index for the children in the sample, represented by the red curve, is shifted to the right when compared to the WHO’s reference curve. This indicates that the majority of the children examined showed tendencies towards excessive malnutrition, specifically overweight and obesity (Figure 3).

### 3.4. Early Childhood Neurodevelopment and Neurocognitive Outcomes

The subscale scores of the MSEL and GGS are shown in Table 3 and Table 4. In our study, the majority (95.8%) of children with SCD had composite T-scores on the MSEL below the average score (T-score < $\bar{x}$ + 2 SD). The mean development quotient was 96.6% for the majority of participants, according to the GGS evaluation standard.

### 3.5. Association Between Early Child Outcomes and Other Covariates

As shown in Table 5 and Table 6, we found a significant association between early child neurodevelopment and maternal depression. The multiple linear regression analysis indicated that, after adjusting for various factors, the scores were significantly related to maternal depression for the MSEL, *p* = 0.03 ddl (N = 7, r = 0.275°), and the GGS, *p* = 0.06 ddl (N = 7, r = 0.244°). Additionally, the MSEL score was related to the Ten-Question Questionnaire (TQQ), *p* = 0.04, and to the age at which the child was weaned from exclusive breastfeeding, *p* = 0.03. The GGS showed a significant association with household socioeconomic status, *p* = 0.04, and the mother’s education level (i.e., years of formal schooling), *p* = 0.01. A significance threshold of α = 0.05 was used for all tests.

## 4. Discussion

Our study is the first to describe the early neurodevelopment and cognitive profiles of children with SCD in the DRC. It highlights the significant impact of SCD on cognition, addresses a key knowledge gap, and informs targeted interventions to improve health outcomes and quality of life.

### 4.1. Child and Maternal Health

In our study, neurodevelopmental delays in walking, language, and adaptation were commonly reported by parents. However, during the neurological examinations, only a few children were identified with neurological sequelae, which may be due to microstroke-related blood flow blockages affecting the brain, potentially resulting in silent cerebral infarctions and persistent neuropsychological deficits, which might be difficult to detect with a standard neurological examination [22]. Although the younger age of participants may explain the lower frequency of neurological sequelae. Our findings align with studies from Cameroon [22] and Congo-Brazza [27], which reported cognitive deficits with age among patients with SCD due to cerebrovascular complications. There is a need for imaging-based tests to measure the impact of SCD on the cerebral level. In resource-limited settings such as the DRC, where imaging is inaccessible for many families, the true prevalence of neurological complications might be underestimated.

### 4.2. Early Child Neurodevelopment and Neurocognitive Outcomes

Our findings show that most of the assessed children had below-average cognitive scores, with fewer than one percent scoring above average. These findings highlight specific neurocognitive deficits among children with SCD in Kinshasa, as well as the need for further research and interventions to address SCD’s impact on development. These observations align with previous research indicating that chronic cerebral hypoxia and silent strokes, common in SCD, significantly contribute to cognitive impairments [4,22]. The cognitive deficits in children with sickle cell disease identified in our study align with previous research. Notably, studies by Bodeau-Livineca F. [21], Boivin [22], and D. Josué Euberma [25] observed similar deficits, although their participants were older than those in our study. Conducting follow-up evaluations as these children transition into adolescence would greatly enhance our understanding of their cognitive development. This proactive approach will enable us to monitor their cognitive progression and address any challenges, ultimately supporting their growth and well-being.

### 4.3. Association Between Early Child Outcomes and Other Covariates

#### 4.3.1. Maternal Depression

Given the impact of maternal depression on child development, there is a need to expand the assessment of depression in mothers at each stage of child development with SCD into old age to determine the impact of this chronic illness on the person’s cognitive function throughout their life. Depression may influence dietary practices, disrupt mother–child relationships, and adversely affect cognitive development. Raising community awareness and providing support systems for mothers and families could alleviate some of these challenges. This associated factor plausibly explains the significant risk of poor early child development in the studied population. It could be argued that the level of maternal depression could potentially lead to lifelong impairment in cognitive function. Maternal depressive symptoms, as reported above, may be explained by the chronic genetic nature of SCD and the socio-cultural context, which may involve interactions between the child, parents, and extended family members concerning the understanding of the disease, as well as conjugal troubles between husband and wife. There is a need for community awareness about this issue to improve the mother’s trust and to support the whole family. Our study is also similar to Itziar Familiar’s study in Uganda [39], where a multivariate analysis showed that maternal depression was an important predictor of reported behaviour scores in children with HIV. This may be explained, firstly, by the chronic nature of both diseases, SCD and HIV, and, secondly, by the similarity in the ages among the studied population. Contrary to a study on Konzo disease, by Esperance Kashala Abotnes, no association was found with maternal depression [24]. SDC is a disease that certainly impacts maternal mental well-being, explaining this maternal depression. There is a need to educate the population on the genetic aspect of the disease, which can be prevented by screening before marriage and increasing awareness about SCD among the population. Bodeau-Livineca F.’s [21] study did not find an association between MSEL and maternal depression, which is likely why we used different tools to evaluate maternal depression. Also, in our study, all participants came from an urban setting, and in their study, they came from a rural setting. In urban settings, parents may be more concerned about their children’s health because they know more about SCD and its impact.

#### 4.3.2. Sociodemographic and Economic Factors

We found that most parents were self-employed. Parents’ socioeconomic background, predominantly self-employed, influenced children’s nutritional status. Wealthier parents tended to provide more consistent care, while poorer families struggled to meet basic dietary needs. Further research is needed to explore how parental occupations and income levels specifically impact child nutrition and to compare SCD children with those affected by other chronic illnesses. There is a need to assess how parental income may affect the nutritional status of children affected by SCD. Our results are similar to Bodeau-Livinec’s study on the MSEL conducted in West Africa [21]. They also found an association between the home environment and the composite score of cognitive development. Both studies used the same tool to evaluate the home environment and cognition. Our research also reinforces Koura’s study in Benin [20] and observations regarding the link between socioeconomic status and child cognitive development, highlighting these factors’ importance in shaping early learning outcomes.

#### 4.3.3. Educational Implications

Many children with SCD in our study lacked access to early education, indicating a significant opportunity for intervention to enhance educational access and opportunities. Many parents hesitated to enroll their children in school, citing financial constraints, fear of stigma due to physical appearance, or a perception that education was unnecessary for children with chronic illnesses. These findings highlight the economic burden of SCD on families and the subsequent impact on children’s educational opportunities and social development. This could explain why some parents are reluctant to spend more money on school fees while they still must pay out of pocket for medical care and will, therefore, prioritize health over education. Our results are, again, similar to those of LIMI Matondo in Tanzania [12]. They observed cognitive function disruption, with lower intelligence quotient (IQ) and altered copy Rey–Osterrieth complex figure (ROCF) scores in SCD children. Although both studies used different tools, our study also found a disturbed neurological function in children with SCD. In addition, school absenteeism was similar for both studies, though the children in Matondo’s study were older in secondary school [12].

#### 4.3.4. Treatment and Cognitive Outcomes

We did not find an association between cognitive function and hydroxyurea’s treatment because fewer children in our study were undergoing treatment with hydroxyurea. Although it is the recommended treatment for SCD, it was, unfortunately, underutilized at the time of the study because of its high cost, despite efforts by the DRC Ministry of Health to improve access. Future research should explore hydroxyurea’s long-term effects on cognitive outcomes in children with SCD. This study reinforces the need for accessible treatment with hydroxyurea, which has shown promise in reducing cerebral complications associated with SCD [40]. Training local healthcare providers in early detection and management of neurocognitive deficits is also crucial.

#### 4.3.5. Maternal Smoking

The smoking statuses of the mothers were not taken into account during our analysis of the data. The practice of smoking is rare among women in the DRC, and, as we expected, most of the selected mothers did not smoke. Only one mother reported smoking during her pregnancy.

#### 4.3.6. Study Strengths and Limitations

Neurocognitive functioning was assessed using well-defined and validated measures, providing a neuro-cognitive profile of young children with SCD in low-income countries. These tests are not invasive and are culturally acceptable. Although we collected data from only three hospitals, patients were selected from all 26 communes of Kinshasa. These hospitals were selected because they offer SCD management in the DRC and receive patients from across the country, as well as neighboring countries.

The robustness of this study was the neurocognitive tests used (MSEL and GGS), which measured cognitive ability and motor development, as well as children’s psychomotor development and growth. These tests are easy to administer and do not require many trained personnel. Also, the tests required less time for a single test, were not invasive, and were culturally acceptable. These factors increased the number of participants who complied with the study during data collection.

The limitations of our study are the lack of a control group and the paraclinical investigations, such as imaging profiles (CT scan and MRI) to evaluate cerebral complications, due to our budget constraints. Our cross-sectional design limits the ability to infer causal relationships. The COVID-19 pandemic prevented us from enrolling more participants. Many parents and caregivers did not bring their children to hospitals, fearing hospital contamination. We, however, followed the Ministry of Health’s COVID-19 prevention protocol to ensure that no one would be infected. None of the studied children were infected during the study. The participants feared taking public transport, which involves proximity to other passengers, increasing the risk of contraction. Consequently, the city’s public transport costs increased as the number of passengers decreased. All of these factors forced us to stop enrolling participants in our study. Finally, another limitation is related to the reduced accessibility of hydroxyurea at the time of the study. Unfortunately, only a few children were treated with it, and this drug was still inaccessible in the country during the study period.

## 5. Conclusions

(1) Only less than one percent of children were above average on the composite T-score, and the majority were below the average score;

(2) The factors associated with neurocognitive profile and early childhood development were maternal depression, physical attitude (TQQ) of the infant, age of food diversification, household socioeconomic level, and the level of the mother’s education.

Our findings highlight the urgent need for early interventions targeting SCD in young children to prevent long-term developmental impairments. This underscores the need to raise public awareness about the disease, improve maternal mental health, and engage national health authorities to better support families living with the burden of SCD. These efforts are essential to enhance the quality of life and cognitive potential of children affected by this chronic illness in the DRC and sub-Saharan Africa.

## 6. Recommendations

Based on the results of our study, we propose the following recommendations to enhance the management and cognitive development of children with SCD in the DRC, organized by domain:

### 6.1. Policy Level

- Launch Awareness Campaigns: initiate awareness campaigns for the public and health professionals concerning SCD and its implications;
- Develop Public Policies: create policies to improve and facilitate access to healthcare for children with SCD and their families;
- Train Healthcare Providers: implement training programs for healthcare providers to effectively detect and manage SCD and its associated neurocognitive deficits;
- Integrate Neurocognitive Screening: incorporate routine neurocognitive screening into care protocols for children with SCD;
- Ensure Accessibility of Hydroxyurea: make hydroxyurea treatment widely accessible across the country;
- Screen for Maternal Depression: recommend systematic screening for maternal depression to support overall family health;
- Implement Psychosocial Support Programs: establish programs to provide psychosocial support for affected families.

### 6.2. Clinical Level

- Facilitate Screening and Diagnosis: organize and enhance access to screening and diagnosis for SCD;
- Strengthen Specialized Care: improve specialized care services, including neurological consultations and early detection of complications;
- Train Health Personnel: provide training for health personnel on managing complications associated with sickle cell disease;
- Ensure Access to Preventive Treatments: guarantee access to preventive treatments, such as hydroxyurea, to minimize the frequency of crises and complications.

### 6.3. Research Level

- Investigate Complications Prevalence: conduct research to better understand the prevalence of complications and their impacts on children with SCD in the DRC;
- Develop Early Identification Strategies: carry out studies to develop strategies for the early identification of neurocognitive deficits in children with SCD;
- Conduct Longitudinal Studies: initiate longitudinal studies to evaluate the impact of SCD on the neurodevelopment of young children;
- Evaluate Community Interventions: assess the impact of community interventions on the management of sickle cell disease and improvements in the quality of life for children with SCD and their families.

## Figures and Tables

**Table 1:** Demographic–economic, maternal, and child characteristics of the 287 studied children aged ≤68 months in Kinshasa, DRC.

	Total
Variable (Unit)	Mean (SD)
Sociodemographic	
Child’s age (years)	3.9 (1.3)
Mother’s age at birth (years)	30.5 (6.9)
Father’s age at birth (years)	38.5 (7.7)
Household size (*n*)	7.5 (2.9)
Number of children ≤ 17 years old in the house (*n*)	3.8 (1.8)
Number of adults > 18 years old in the house	3.8 (1.9)
Number of siblings (*n*)	3.0 (1.6)
Rank of the child (*n*)	2.8 (1.6)
Monthly income (Congo Francs)	347,379.6 (278,702.1)
Maternal characteristics	
Hopkins Symptoms Checklist (scores)	8.5 (5.4)
Child characteristics	
Birthweight (grams)	3274.8 (552.2)
Child’s weaning age (months)	5.6 (0.8)
TQQ * (scores)	3.9 (1.2)
Height (cm)	102.7 (17.3)
Head circumference (cm)	50.6 (1.9)
Number of meals the previous day (*n*)	1.5 (0.6)
Socioeconomic characteristics N (%)	
Father’s occupation	
Self-employed	150 (52.3)
Public servant	61 (21.3)
Privately employed	48 (16.7)
Unemployed	28 (9.8)
Mother’s occupation	
Self-employed	140 (48.8)
Public servant	107 (37.3)
Privately employed	22 (7.7)
Unemployed	18 (6.3)
Wealth index: household income level **	
Lowest tertile	96 (33.4)
Average tertile	95 (33.1)
Highest tertile	96 (33.4)

* TQQ: Ten-Question Questionnaire. ** Wealth index. Households were ranked in ascending order according to the total score and divided into three categories of equal size, called tertiles.

**Table 2:** Parental level of education and parenting style (stimulation & learning opportunities) of the 287 studied children with SCD aged ≤ 68 months in Kinshasa, DRC.

	Total	
Variable	Effective	Percentage, %
Maternal factors		
Maternal smoking during pregnancy	4	1.4
Maternal alcohol use during pregnancy	81	28.2
Mother’s level of education		
Primary	24	8.4
Secondary	156	55.1
Professional	51	17.8
University	54	18.8
Father’s level of education		
Primary	1	0.3
Secondary	89	31.0
Professional	23	28.9
University	114	39.7
Stimulation, learning opportunities, and parenting style		
Storytelling to kids	167	58.2
Uses books with pictures	130	45.3
Reads to the child	129	44.9
Plays with kitchenette	263	91.6
Plays with homemade toys	229	79.8
Plays with bought toys	274	95.5
Left home alone and unsupervised by an adult more than 10 times/month	9	3.1
Explains without being upset	133	46.3
Raises voice	192	66.9
Takes away toys as punishment	145	50.5
Gives extra work as punishment	52	18.1
Spanks to punish	217	75.6
Uses stick to punish	11	3.8
Hits on the head to punish	23	8.0

**Table 3:** Proportion of children with SCD who had composite scores on the MSEL.

	Effective	%
Above	11	3.83
Average	1	0.35
Below	275	95.82
Total	287	100.00

**Table 4:** Mullen Scales of Early Learning and Gensini Gavito Scale scores for the 287 studied children aged 0–68 months old in Kinshasa, Democratic Republic of Congo.

	Participants
Variable	N = 287
1. Mullen Scales of Early Learning (N = 43)	
T-scores: gross motor	
Mean (SD)	44.67(13.20)
T-scores: visual reception	
Mean (SD)	53.09 (218.93)
T-scores: fine motor	
Mean (SD)	33.83(12.23)
T-scores: receptive language	
Mean (SD)	44.38(10.06)
T-scores: expressive language	
Mean (SD)	51.45(11.86)
T-scores: cognition	
Mean (SD)	171.81(33.92)
2. Gensini Gavito Scale	
Child developmental age (years)	
Mean (SD)	3.87(1.26)
Child developmental quotient (%)	
Mean (SD)	96.59(12.54)

TSGM: t-core gross motor, TSVR: t-score visual reception, TSFM: t-score fine motor, TSRL: t-score receptive language, TSEL: t-score expressive language.

**Table 5:** Factors associated with neurodevelopment and cognition in children with SCD (Mullen Scales of Early Learning).

	Non-Standardized Coefficients		Standardized Coefficients	t	Sig.	95.0% Confidence Interval for B		Collinearity Statistics	
Variables	Beta	Standard Error	Beta			Lower Bound	Upper Bound	Tolerance	VIF
(Constant)	182.342	21.197		8.602	0.000	140.616	224.068		
Nutrition	−0.694	0.442	−0.097	−1.571	0.117	−1.564	0.176	0.867	1.153
Food safety	−1.291	0.661	−0.128	−1.954	0.052	−2.593	0.010	0.775	1.291
Education, educational style, and environment	0.607	0.549	0.070	1.106	0.270	−0.474	1.689	0.828	1.207
Reaction last 12 months	2.197	1.555	0.084	1.413	0.159	−0.864	5.257	0.943	1.060
Hopkins Symptoms Checklist (HSCL-10) Maternal depression	−0.837	0.380	−0.134	−2.201	0.029	−1.586	−0.088	0.899	1.112
TQQ scores (10 Questions)	5.006	1.715	0.172	2.919	0.004	1.630	8.383	0.958	1.044
Socioeconomic level	−0.453	0.665	−0.044	−0.681	0.496	−1.761	0.856	0.789	1.267
Type of breastfeeding received at birth	3.579	6.914	0.031	0.518	0.605	−10.032	17.189	0.950	1.052
Age of food diversification	−5.535	2.572	−0.129	−2.152	0.032	−10.598	−0.473	0.942	1.061
Total ponderation of risk factors for early cerebral morbidity	−0.113	0.168	0.018	−0.672	0.502	−0.444	0.218	0.962	1.014
Mother’s level of education	2.776	2.359	0.078	1.177	0.240	−1.868	7.420	0.776	1.288
Father’s level of education	−0.541	2.670	−0.014	−0.203	0.839	−5.797	4.714	0.760	1.316

**Table 6:** Factors associated with neurodevelopment and cognition in children with SCD (Gensini Gavito).

	Non-Standardized Coefficients		Standardized Coefficients	t	Sig.	95.0% Confidence Interval for B		Collinearity Statistics	
Variables	B	Standard Error	Beta			Lower Bound	Upper Bound	Tolerance	VIF
(Constant)	90.165	7.890		11.428	0.000	74.634	105.696		
Food safety	0.070	0.246	0.019	0.284	0.776	−0.414	0.554	0.775	1.291
Education, educational style, and environment	0.096	0.204	0.030	0.469	0.640	−0.307	0.498	0.828	1.207
Hopkins Symptoms Checklist (HSCL-10) Maternal depression	−0.390	0.142	−0.169	−2.753	0.006	−0.668	−0.111	0.899	1.112
TQQ (10 Questions)	0.532	0.638	0.050	0.833	0.405	−0.725	1.789	0.958	1.044
Socioeconomic level	0.512	0.247	0.136	2.071	0.039	0.025	0.999	0.789	1.267
Nutrition	−0.203	0.164	−0.077	−1.237	0.217	−0.527	0.120	0.867	1.153
Reaction last 12 months	0.135	0.579	0.014	0.233	0.816	−1.004	1.274	0.943	1.060
Type of breastfeeding received at birth	−0.231	2.475	−0.005	−0.093	0.926	−5.104	4.642	0.950	1.052
Age of food diversification	−0.267	0.921	−0.017	−0.290	0.772	−2.080	1.545	0.942	1.061
Total ponderation of risk factors for early cerebral morbidity	0.086	0.125	0.037	0.690	0.495	−0.160	0.332	0.962	1.040
Mother’s level of education	2.255	0.845	0.170	2.670	0.008	0.592	3.917	0.776	1.288
Father’s level of education	−1.011	0.956	−0.068	−1.058	0.291	−2.893	0.870	0.760	1.316

## Data Availability

Data that support the reported results can be requested from the corresponding author upon reasonable request due to privacy.
